# Merging game theory and control theory in the era of AI and autonomy

**DOI:** 10.1093/nsr/nwaa046

**Published:** 2020-03-16

**Authors:** Ming Cao

**Affiliations:** Faculty of Science and Engineering, University of Groningen, The Netherlands

## Abstract

Game theory and control theory, each powerful in its own right, can nourish each other in the focused area of intelligent and autonomous decision-making processes. In fact, the two theories enhancing each other is a must in response to the opportunity and need to design and implement AI and autonomy.

The recent enthusiasm among the general public on artificial intelligence (AI) and autonomous robots, evidenced by vigorous discussions on social media, deserves applause for igniting passion for conceptualizing futuristic technological development, and consequently for bringing closer society's curiosity and scientists’ pursuit. However, as the discussions become increasingly involved in various sectors, as scientists and engineers, we ourselves must be more engaged in building a research roadmap and, in particular, innovate mathematical tools in efforts to develop a rigorous theoretical framework for research. Among the wide range of discussed topics, one particularly scrutinized topic is how AI might replace humans’ making decisions in daily lives. Game theory and control theory in combination are key in this context due to their central role in understanding decision-making in a dynamically changing world.

## GAME THEORY AND CONTROL THEORY IN A NUTSHELL

Modern game theory studies how decisions made by different entities or actors (termed players) affect each other. It was formalized by mathematician John von Neumann and economist Oskar Morgenstern, who together published the ground-breaking book *Theory of Games and Economic Behavior* [1]. In the late 1940s and early 1950s, the mathematician John Nash showed that, in a game in which each rational player chooses a strategy taking into account how the other players may choose their strategies, a ‘Nash equilibrium’ occurs when no player is able to improve her own situation by unilaterally changing strategy; with mathematical insight, Nash revealed that every game with a finite number of players, each with a finite number of candidate strategies, has at least one such equilibrium [2]. Inspired by the study of animal behaviors, biologist John Maynard Smith gave a twist to classical game theory by looking for ‘evolutionarily stable strategies’ that are stable outcomes in populations of players undergoing evolutionary games mimicking natural selection [3]. Since AI and autonomy complicate the profiles of decision makers, the notions of equilibria and long-run stable strategies in game theory can anchor the analysis and prediction of complex decision-making dynamics.

Control theory is concerned with introducing control actions into a dynamical system to ensure the system's stability. In the 1950s, ‘dynamic programming’ [4] and ‘maximum principle’ [5], developed by mathematicians Richard Bellman and Lev Pontryagin, respectively, led to the accelerating development of optimal control theory that is aimed at finding optimal control actions over a period of time for a given objective function and has found quickly numerous applications in both science and engineering [6]. Control theorists noticed early on the possibility of merging game theory with control theory, and made an original contribution in formulating and analysing dynamic games that focus on multiple players dynamically updating their decisions over time, who may have completely different cost functions and knowledge of the game [7]. Because learning algorithms, especially multi-agent reinforcement learning algorithms, are now enabling machines to outperform humans in some complex environments [8], the key ideas of feedback (more general than reinforcement) and optimality from control theory may become instrumental in both model-based and model-free approaches to learning.

## FURTHER DEVELOPING GAME THEORY NEEDS CONTROL THEORY AND VICE VERSA

Game theory and control theory, each powerful in its own right, can nourish each other much more in the focused area of intelligent and autonomous decision-making processes, which are becoming the most critical components in a growing number of natural, social and engineered large-scale systems; in fact, the two theories enhancing each other is a must in response to the opportunity and need to design and implement AI and autonomy. One salient enhancement starting to take shape recently is to introduce dynamic incentives as feedback in order to tackle the ‘price of anarchy’ for groups of self-interested individuals. The self-enforcing Nash equilibria and evolutionarily stable strategies, often sub-optimal or even associated with the worst social benefits, helped economists and biologists alike to understand that self-improving individuals can lead to self-harming groups. In such situations, individuals need to be incentivized to be guided to better other equilibria, if not the best. The design and testing of such feedback can be challenging in real life due to individuals’ partial knowledge of the whole system, changing network structures in the population, dynamic and uncertain environments and potential conflict with self-contained AI algorithms. Incentive-based control for games has huge potential to grow to address how to reach social optimality in collective decision-making [9].

**Figure 1.**
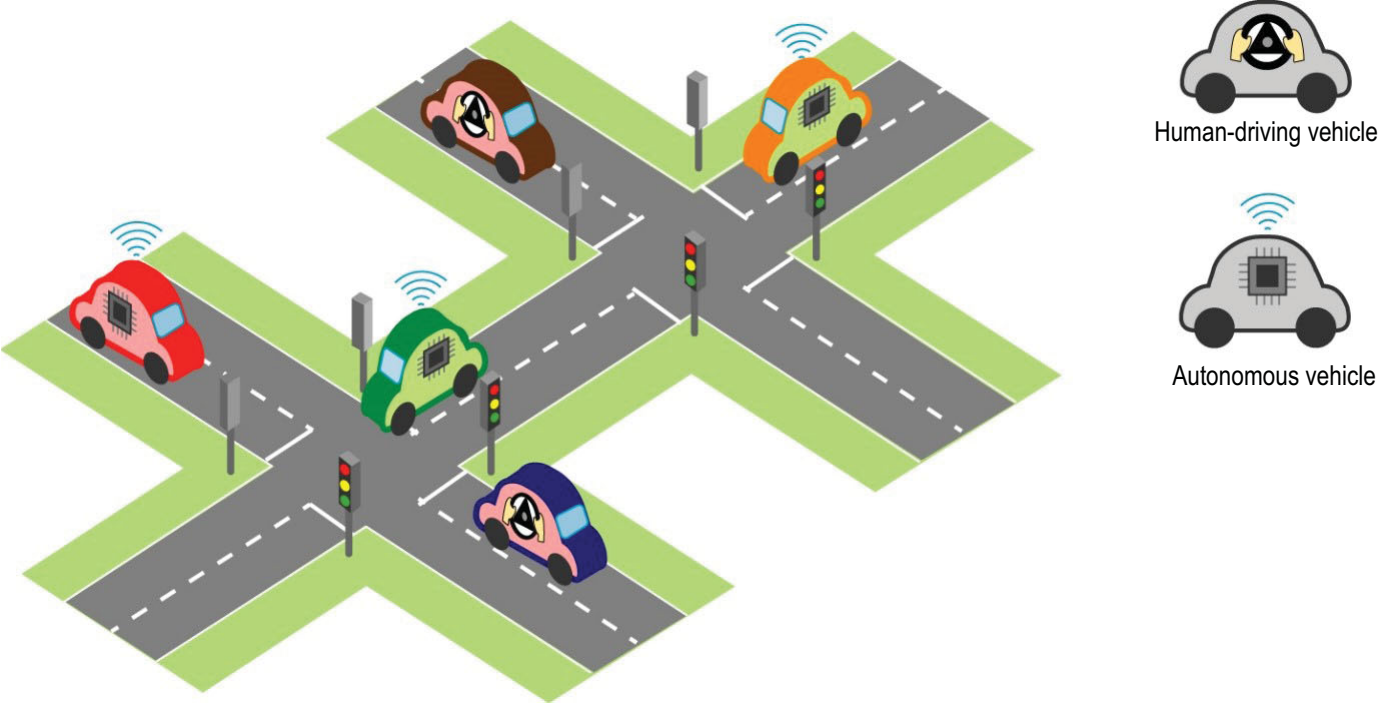
Managing traffic with mixed human-driving and autonomous vehicles requires analysing decision-making individuals in cyber-physical-social systems.

A second enhancement still in its early stage is to formally consider cognitive characteristics of decision-making individuals in cyber-physical-social systems, especially those with the components of human-in-the-loop control systems. Experience makes players wiser; close-loop makes controllers stabilizers; and to develop a wiser stabilizer in large-scale complex systems involving complicated intertwined decision-making processes requires going beyond the existing often overly simplified game models and human-in-the-loop control system models. One example in point is traffic systems (Fig. 1): it is well known in game theory that self-interested drivers choosing the quickest route may worsen everyone's choices and lead to traffic jams. Introducing autonomous vehicles guided by advanced control algorithms itself will not solve the problems if people's social norm and habits on the road are not taken into account and a future intelligent traffic system is only feasible when control adapts to cognitive decision-making drivers, human or non-human.

Better dealing with an uncertain future is another enhancement that can be achieved by jointly exploiting game theory's strategic prediction and control theory's convergence analysis. Discounting future payoffs without knowing for sure when the task can be accomplished is already tricky for a small team, let alone to consider similar calculations in large interacting populations [10]. Consider again an example. A human–robot team is carrying out a search-and-rescue task in the wild under communication constraints. Each autonomous robot needs to adjust its searching behavior according to its belief on how likely a survivor can be found in the near or far future while reasoning about its robotic or human peers’ intention and ability to continue searching. To sustain cooperation, each robot must be able to reliably predict locally how group behavior converges and how future gains and losses can be properly discounted for the present to optimize its current strategic decision.

## OUTLOOK

The three major enhancements just discussed of game theory and control theory all contribute to an overarching ambitious goal to integrate learning, optimization and control for intelligent and autonomous complex networks and systems. Such a goal has never been more tantalizingly achievable given the breakthroughs in AI and autonomy. To reach this goal and judging from the accumulated knowledge and ongoing explosive research efforts in game and control theories, we don’t have to wait long!

***Conflict of interest statement.*** None declared.
